# Vanishing twins, selection *in utero*, and infant mortality in the United States

**DOI:** 10.1093/emph/eoae035

**Published:** 2025-01-08

**Authors:** Ralph Catalano, Joan Casey, Allison Stolte, Hedwig Lee, Alison Gemmill, Brenda Bustos, Tim Bruckner

**Affiliations:** School of Public Health, University of California, Berkeley, CA, USA; Department of Environmental and Occupational Health Sciences, University of Washington School of Public Health, Seattle, WA, USA; Department of Health, Society, and Behavior, Joe C. Wen School of Population and Public Health, University of California, Irvine, CA, USA; Department of Sociology, Duke University, Durham, NC, USA; Department of Population, Family, and Reproductive Health, Johns Hopkins Bloomberg School of Public Health, Baltimore, MD, USA; Department of Health, Society, and Behavior, Joe C. Wen School of Population and Public Health, University of California, Irvine, CA, USA; Department of Health, Society, and Behavior, Joe C. Wen School of Population and Public Health, University of California, Irvine, CA, USA

**Keywords:** Vanishing Twin Syndrome, Infant Mortality

## Abstract

**Background and Objectives:**

Research to identify fetal predictors of infant mortality among singletons born in the United States (US) concludes that poorly understood and unmeasured “confounders” produce a spurious association between fetal size and infant death. We argue that these confounders include Vanishing Twin Syndrome (VTS)—the clinical manifestation of selection against frail male twins *in utero*. We test our argument in 276 monthly conception cohorts conceived in the US from January 1995 through December 2017.

**Methodology:**

We use Box-Jenkins transfer function modeling to test the hypothesis that among infants born from 276 monthly conception cohorts conceived in the US from January 1995 through December 2017, the sex ratio of twins born in the 37th week of gestation will correlate inversely with infant mortality among singleton males born at the 40th week of gestation.

**Results:**

We find support for our hypothesis and infer that the contribution of survivors of VTS to temporal variation in infant mortality among the hardiest of singleton male infants, those born at 40 weeks gestation, ranged from a decrease of about 7% to an increase of about 5% over our 276 monthly conception cohorts.

**Conclusions and Implications:**

We conclude that an evolutionary perspective on fetal loss makes a heretofore “unmeasured confounder” of the relationship between fetal size and infant mortality both explicable and measurable. This finding may help clinicians better anticipate changes over time in the incidence of infant mortality.

## INTRODUCTION

Scholars have long suggested that conception endows a human fetus with a unique “schedule” for development *in utero* [1, 2]. Absent chromosomal and genetic defects, and assuming sufficient maternal investment, pregnancy presumably progresses as scheduled and yields an infant hardy enough to survive in the prevailing environment [3].

The above argument intuitively implies that two circumstances can preclude scheduled development. First, parturition before completing the schedule can yield an infant ill-prepared for the prevailing environment. Epidemiology has confirmed this intuition in that early birth remains a strong predictor of death in the first year of life [4].

Second, underinvestment of maternal resources perturbs gestation during a critical interval leaving the fetus without some fraction of otherwise scheduled development [5, 6]. Gestation continues to birth but an anomalous developmental sequence puts the infant at risk of death early in life [1–3].

Clinicians can identify an infant put at increased risk of death by early parturition because they observe the length of gestation. But infants at risk due to a developmental anomaly remain difficult to identify because clinicians cannot compare observable indicators of fetal development to an unobservable schedule. Based on the assumption of similarity among endowed schedules, researchers have provided an intuitively appealing alternative by constructing statistical distributions of fetal size at various gestational ages [7, 8]. Clinicians and epidemiologists have speculated that developmental anomalies put fetuses near the bottom of these distributions. Tests of this speculation have found that although “small for gestational age” (i.e. SGA) fetuses (i.e. those below the 10_th_ percentile of the distribution of size for gestational age) disproportionately suffer infant morbidity and mortality, most appear simply “constitutionally small” in that they do not exhibit lethal pathologies at a rate greater than other infants [9].

Epidemiologists have searched for indicators, other than SGA, that might identify fetuses with potentially fatal developmental anomalies, but no compelling alternative has emerged [10]. They, therefore, refer to pathological developmental anomalies as unmeasured “confounders” that cause infant mortality and appear disproportionately among SGA fetuses [11, 12]. Here we argue that Vanishing Twin Syndrome (i.e. VTS) qualifies as such a confounder because it yields small singleton infants at risk of infant death due to developmental anomalies. We also argue that an evolutionary perspective on fetal loss would lead clinicians to anticipate that VTS would confound the association between small fetal size and infant mortality. We furthermore argue that researchers can measure, albeit indirectly, the contribution of VTS to variation in infant mortality among conception cohorts. We test our arguments using 276 monthly conception cohorts begun in the United States (i.e. US) from January 1995 through December 2017.

With rare exceptions [12, 13], research seeking indicators of developmental anomalies in fetuses has not included twins probably because they represent fewer than 3% of newborns and because their gestations appear constrained by circumstances different from those constraining singletons. The most obvious of these constraints include those on maternal investment. Twin fetuses must share maternal resources “sized” to support the development of a singleton [14]. Consistent with the possibility that sharing these resources increases the risk of missing critical windows of development, twins not only appear disproportionately among SGA infants [15] but also exhibit relatively high rates of fetal mortality compared to singletons [16–19]. Furthermore, and consistent with pathologically anomalous development, among infants born “term” (i.e. after 37 complete weeks of gestation), twins are more likely to die in the first year of life than singletons. In the US, for example, term male twins die at a rate (i.e. 4.56 per 1000) 81% higher than term male singletons (2.52 per 1000) [20].

Fetuses in a twin pair typically grow at different rates. Twin sets exhibiting relatively great discordance in size disproportionately suffer VTS in which the smaller twin dies *in utero* [21]. Estimates of the frequency of VTS reach as high as 36% of twin sets [22]. If the fetus that does not vanish survives to birth, vital statistics systems will register the infant as a singleton. Singleton survivors of VTS, however, suffer infant mortality at a rate similar to twins and, therefore, higher than the rate among true singletons [23, 24].

Clinical research into the sequelae of VTS and into fetal predictors of infant mortality has proceeded separately despite the theoretical and empirical connection between them. We suggest that this connection becomes intuitive, if not obvious, if researchers view gestation as much an opportunity for selection as for maturation. VTS then appears as one of several mechanisms collectively referred to as “selection *in utero*” [25]. These mechanisms presumably arise from mutations conserved by natural selection because they abort fetuses least likely, if born, to thrive in the prevailing environment [26–29]. Gestations of male twins are thought to be at particularly high risk of selection *in utero* because they have historically produced fewer grandchildren than gestations of female twins or of singletons [30–32]. The relatively low reproductive fitness of male twins presumably arises because in virtually every society and year for which we have dependable vital statistics, male infants remain the human most likely to die before the end of reproductive life [33]—and, as noted above, male twins more likely die in infancy than do male singletons. Consistent with the inference that high mortality among male twins causes their low reproductive fitness, the disparity in fitness between them and other offspring appears greatest in populations occupying environments threatening to frail infants [32].

Males who gestate as twins and who die in infancy, which they do at a higher rate than other male infants [34, 35], appear in the “ledger” of vital statistics as either twins or singletons depending on whether the other fetuses in their gestations “vanished.” This implies that infant mortality among males should vary positively over conception cohorts with the frequency of male VTS survivors in the cohort. As described below, we aim to test this reasoning in 276 monthly conception cohorts begun in the US from January 1995 through December 2017.

Impediments to converting the above reasoning into a testable hypothesis include that researchers cannot know the true frequency of VTS in conception cohorts. Much, if not most, vanishing likely occurs before clinical recognition of pregnancy. Although records kept after recognition may include notice that a twin had vanished, no one to our knowledge has drawn a representative sample of obstetric records in the US to estimate the frequency of VTS in conception cohorts. Fetal deaths in the US, moreover, are typically registered, with questionable accuracy, only after the 20th week of gestation. These registrations, furthermore, do not routinely report whether the demise was of a fetus in a twin set [36]. Nor do birth certificates of singletons note that the infant had survived VTS. These circumstances imply that we can only indirectly gauge the frequency of survivors of VTS among males born from monthly conception cohorts.

We attempt to overcome the above impediment by using the ratio of male to female twins born live in the 37th week of gestation as our indicator of the frequency of survivors of VTS among singleton males born from monthly conception cohorts. This indicator assumes that VTS, as a manifestation of selection *in utero*, “culls” the frailest male twin fetuses in a conception cohort thereby converting a subset of twin to singleton gestations. The “tolerance” of culling for fetal frailty will vary over conception cohorts with stressors on the population [37] and with characteristics of the pregnant persons [38, 39] contributing to the cohorts. We reason that “deep” culling will convert relatively many twins to singleton gestations in a conception cohort. This implies that the remaining twin sets will have relatively few males. The conversion of relatively many twins to singleton gestations also implies, however, that singletons born from the cohort will include relatively many male survivors of VTS.

We use the sex ratio of twins born in the 37th week of gestation because more twins are born in that week [20] than in any other implying that frailty due to early or late birth, rather than developmental anomalies, will appear low among them compared to other twins.

A second impediment to converting the above argument into a testable hypothesis arises from the facts that preterm (i.e. before 37 complete weeks of gestation) and post-term birth increase the risk of infant mortality [4] and that survivors of VTS appear disproportionately among early births [40]. An association between an indicator of the frequency of survivors of VTS among male infants born from monthly conception cohorts and of singleton male infant mortality in those cohorts could, therefore, arise solely from early birth.

We overcome the above impediment by restricting our dependent variable, male infant mortality in conception cohorts, to singletons born in the 40th week of pregnancy (i.e. the week of gestation when most singletons are born). This restriction “controls” the potentially confounding effect of gestational age at birth on infant mortality because all members of the test population were born at the same gestational age.

We test the hypothesis, implied by the anomalous development argument, that the ratio of male to female twins born in the 37th week of gestation from monthly conception cohorts will correlate inversely with the frequency of infant mortality among male singletons born in 40^th^ week of gestation from the same cohorts.

## METHODS

### Data

We used 1995–2019 restricted-use period linked birth-infant death data from the National Center for Health Statistics. These data include information on the plurality, gender, gestational age, and birth month/year of each birth and death. Our analyses excluded the births (0.18%) and deaths (0.11%) of infants whose mothers lived outside of the US at the time of birth. The University of California, Irvine Committee for the Protection of Human Subjects approved this study (protocol # 20195444).

We estimated conception cohorts using the birth month and year of each birth/infant death. Because the restricted-use data do not include the day of birth, we first randomly assigned the births/infant deaths to a day within their given birth months. We then used last menstrual period, the only continuously reported measure of gestational age during the study period [41], to estimated gestational age at birth in days (i.e. gestational weeks x 7 days). Finally, we subtracted gestational days from the randomly assigned date of birth to estimate the conception date. In our analysis, we used conception *month*, which smoothed small, random errors in conception dates that resulted from the random assignment of the day of birth.

Information on births and infant deaths included birth month and year. About 0.5% of all births (*N* = 518 680) and 1.93% of all deaths (*N* = 12 428) were missing gestational age, such that we could not estimate conception month. We used those births/deaths to infants conceived between January 1995 and December 2017, which allowed enough time for infants in each cohort to develop *in utero* to 40 weeks gestation and survive through their first year of life, ensuring that we had complete infant survival/death data in our analysis. The data for our analysis included births of twins born at 37 weeks gestation (*N* = 521 692) and births/deaths of singleton infants born at 40 weeks gestation (*N* = 18 353 850 births; *N* = 35 117 deaths).

Using eligible births/deaths, we aggregated counts of births and infant deaths for each conception cohort by gender and plurality (no missingness). Importantly, the gender- and plurality-specific counts of births and deaths indicate those births and deaths to infants *conceived* in the given monthly conception cohort.

### Statistical analyses

We tested our hypothesis through the following steps.

1. We regressed the number of infant deaths among male singletons born at 40 weeks gestation from 276 monthly conception cohorts begun in the US from January 1995 through December 2017 on the number of males (in 1000s) born from the cohorts at 40 weeks gestation, and, as a negative control, on the number of infant deaths among female singletons born at 40 weeks gestation. This step controls for the size of the population at risk as well as for confounders that affect risk of infant death regardless of sex.
2. We used Box and Jenkins [42] methods to identify and model autocorrelation in the residuals of the Step 1 regression. These methods not only detect autocorrelation, but also determine which of a very large family of models best fits it. Box and Jenkins methods attribute strong cycles (e.g. seasonality in monthly measurements) and linear trends to integration while attributing the tendency for high or low values to persist into subsequent measurements (i.e. regression to expected) to either moving averages (for relatively short persistence) or autoregression (for relatively long persistence). Well-developed and widely used rules refined over decades of use in the sciences [43], including epidemiology [39], determine which combination of these three types of parameters a researcher needs to “fit” a series. Step 2 yields a Box-Jenkins “transfer function” that models deaths among singleton males born in the 40^th^ week of gestation from monthly conception cohorts as a function of all singleton males born at 40 weeks from the cohorts, infant deaths among females born at 40 weeks from the cohorts, and autocorrelation. The residuals of this model, which are independent of each other and have a mean of 0, gauge the degree to which infant mortality among singleton males born at 40 weeks gestation from monthly conception cohorts differed from statistically expected values.
3. We tested our hypothesis by estimating the Box-Jenkins transfer function formed by expanding the transfer function developed in Step 2 to include the ratio of male to female twins born in the 37th week of gestation. We infer support for our hypothesis that survivors of VTS contribute to temporal variation in singleton male infant mortality if the estimated coefficient for the ratio and the bounds of its 95% confidence interval, appear negatively signed.

## RESULTS

**Figure 1.**
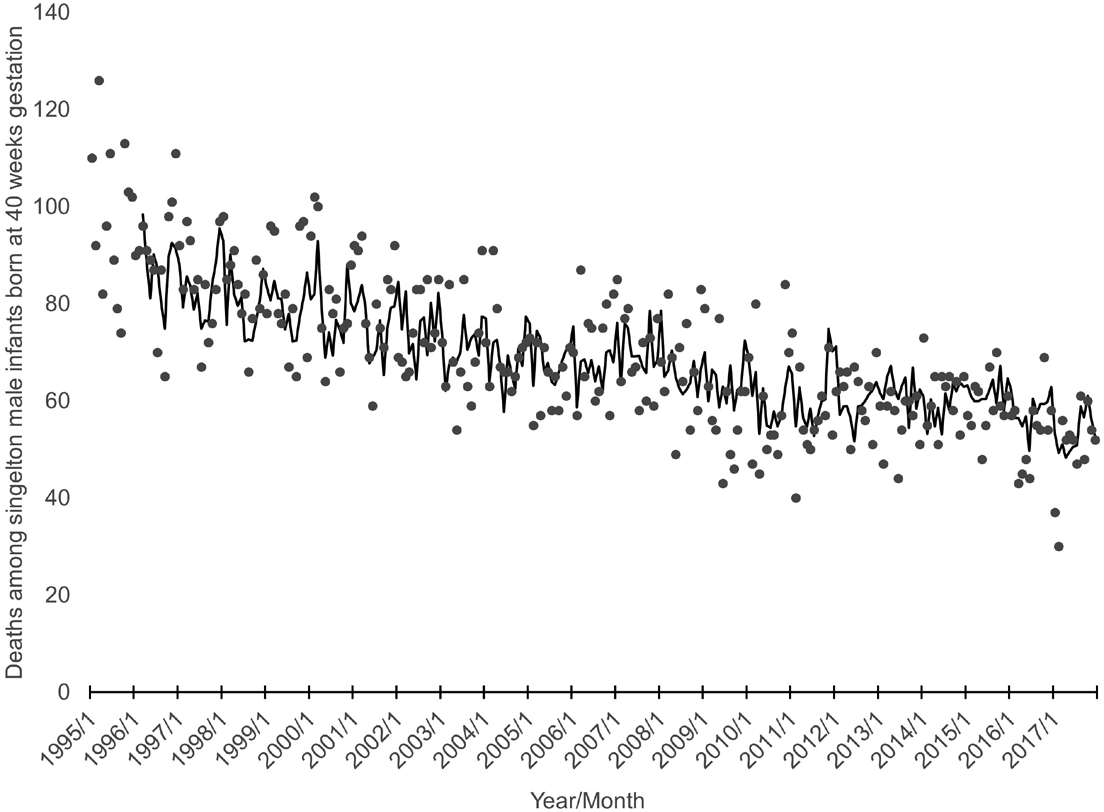
Observed (points) and expected (line: 14 cases lost to modeling) deaths among singleton male infants born at 40 weeks gestation from 276 monthly conception cohorts begun in the US from 1/1995 through 12/2017.

Our dependent variable, infant deaths among male singletons born at 40 weeks gestation in the US, ranged from 30 to 126 with a mean of 69 over 276 conception cohorts beginning January 1995 and ending December 2017. Figure 1 shows the deaths, as points, plotted over time.

**Figure 2.**
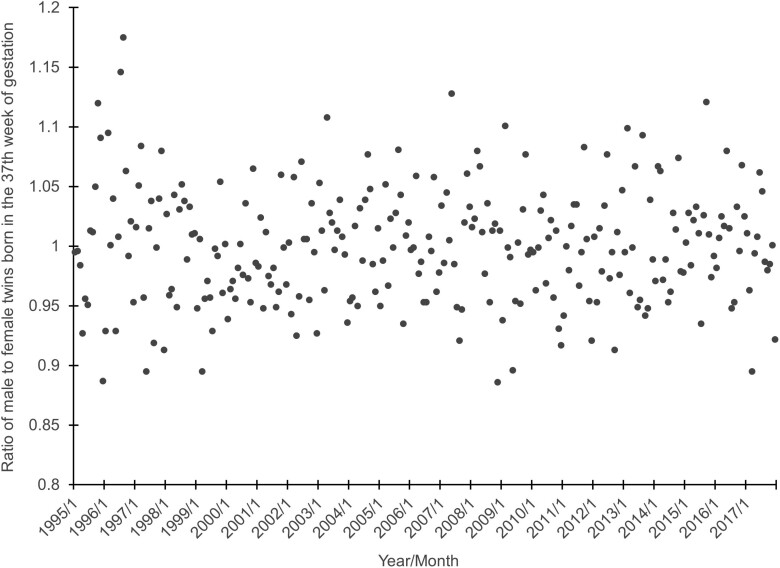
Ratio of male to female twins born at 37 weeks gestation from 276 monthly conception cohorts begun in the US from 1/1995 through 12/2017.

The ratio of male to female twins born at 37 weeks gestation ranged from 0.89 to 1.18 with a mean of 0.99 over the 276 conception cohorts. Figure 2 shows the ratio, as points, plotted over time.

Results from Steps 1 and 2, in which we used Box-Jenkins methods [37] to model infant deaths among male singletons born at 40 weeks gestation as a function of males born (in 1000s) from the cohorts at 40 weeks gestation, infant deaths among females born from the cohorts at 40 weeks, and autocorrelation yielded the following transfer function in which all estimated coefficients were at least twice their standard errors.


$$\begin{array}{lll} Y_{t}= & \;1.76X_{1t}+0.16X_{2t}+(1+0.21B^{10})/(1-0.13B)\; & \;(1-0.31B^{12}-0.26B^{13})e_{t} \end{array}$$

Y_t_ is infant deaths among male singletons born at 40 weeks of gestation in month t. X_1t_ is male singleton births (in 1000s) at 40 weeks gestation in month t. X_2t_ is infant deaths among female singletons born at 40 weeks of gestation in month t. 0.22B^10^ is a moving average parameter connoting that high or low values in the residuals tend to “echo,” albeit diminished, 10 months later. –0.13B is an autoregressive parameter showing that high or low values of Y persist, although diminished, into the next month. –0.32B^12^ and –0.25B^13^ are seasonal autoregressive parameters suggesting that values of Y at time t predict those a year later. e_t_ is the residual that measures the difference between the expected and observed Y at month t. These residuals exhibit no autocorrelation and have a mean of 0. Figure 1 shows, as a line, the statistically expected value of infant deaths among male singletons born at 40 weeks of gestation.

In Step 3, we tested our hypothesis by estimating the Box-Jenkins transfer function [42] formed by adding the ratio of male to female twins born in the 37th week of gestation, which exhibited no autocorrelation, to the transfer function identified in Step 2. The results of that estimation were as follows in which all coefficients were at least twice their standard errors.


$$\begin{array}{lll} Y_{t}= & \;2.57X_{1t}+0.17X_{2t}-27.29X_{3t}\; & +\left(1+0.23B^{10}\right)/\left(1-0.28B^{12}-0.29B^{13}\right)e_{t} \end{array}$$

Y_t_, X_1t_, X_2t_, and e_t_ are as noted in Steps 1 and 2. X_3t_ is the ratio of male to female twins born in the 37th week of gestation. The estimated coefficient for X_3_ (i.e. –27.29), and the bounds of its 95% confidence interval (i.e. –43.10; –11.47) were negatively signed. We, therefore, infer support for our hypothesis and for our argument that survivors of VTS contribute to singleton male infant mortality.

**Figure 3.**
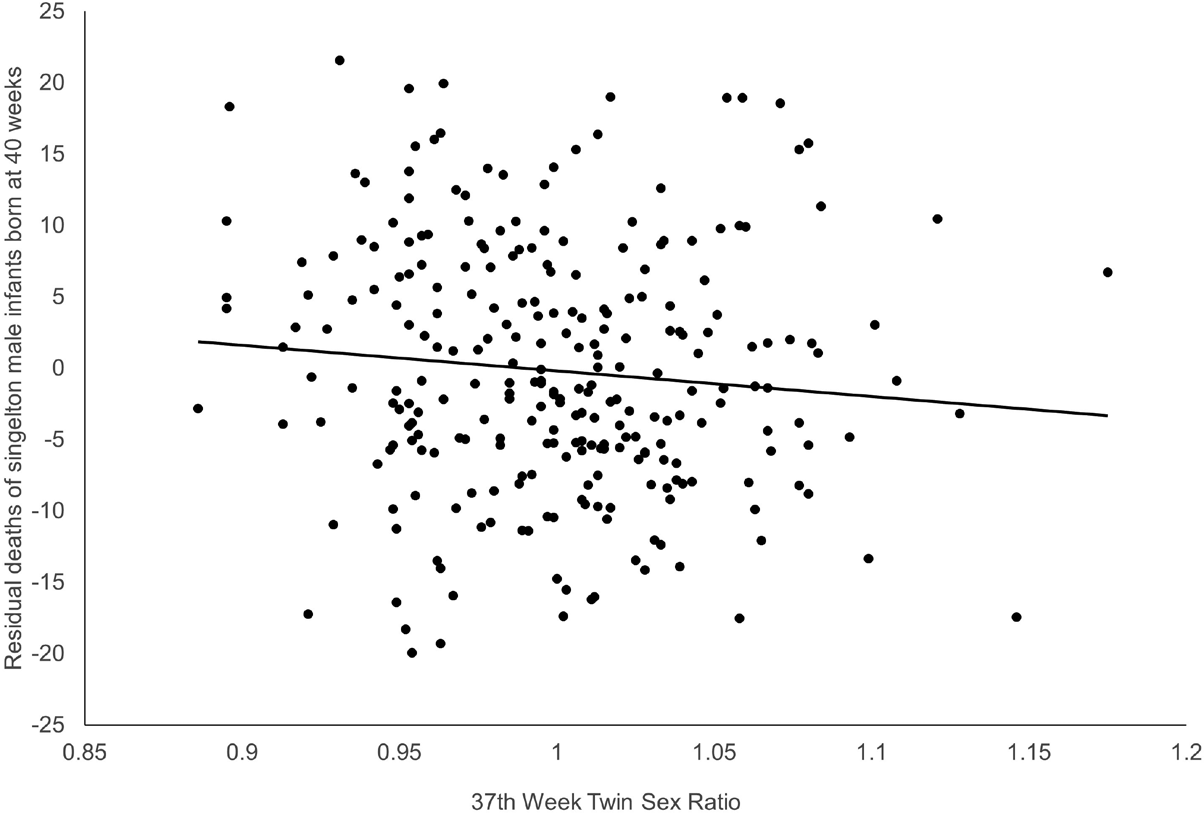
Scatter plot of residual deaths among singleton male infants born at 40 weeks gestation and the M/F sex ratio of twins born at 37 weeks gestation. *N* is 276 monthly conception cohorts begun in the US from 1/1995 through 12/2017.

Figure 3 shows a more graphic presentation of our findings. The scatter plot shows the residuals of the model estimated in Step 2 over the M/F sex ratio of twins born at 37 weeks gestation. The downward slope of the regression line reflects the coefficient for X_3_ (i.e. –27.29) reported above.

Following the convention [43] of assessing the sensitivity of time-series tests to specification of autocorrelation in the dependent variable, we repeated our test but substituted the “second best” Box-Jenkins model for that we identified in Step 2. “Second best” refers to the Box-Jenkins model that, among those that remove autocorrelation from the dependent series, yields the second lowest Akaike Information Criterion score. Rules [42] for identifying autocorrelation found only two models that fit our dependent series. The second-best model fit seasonality by differencing the series at 12 months (i.e. subtracting the value at time t from that at t + 12) rather than by specifying autoregression at t + 12 and t + 13 as in the best-fitting model. The results of the alternative test converged with those of our main test. The estimated coefficients, all of which were at least twice their standard errors, were as follows.


$$\begin{array}{lll} \Delta_{12}Y_{t}= & \;2.66\Delta_{12}X_{1t}+0.13\Delta_{12}X_{2t}\; & -1.19X_{3t}+\left(1+0.14B^{10}\right)\left(1-0.67B^{12}\right)e_{t} \end{array}$$

Y_t_, X_1t_, X_2t_, X_3t_, and e_t_ are as noted above. The estimated coefficient for the ratio of male to female twins born in the 37th week of gestation (i.e. –1.19), and the bounds of its 95% confidence interval (i.e. –1.76; –0.62), were negatively signed.

We performed additional calculations to provide a sense of the contribution of survivors of VTS to temporal variation in infant mortality among male singletons born at 40 weeks gestation. First, we took the first differences (i.e. value at t subtracted from that at t + 1) of our dependent variable (i.e. the ratio of male to female twin births at 37 weeks gestation) thereby allowing us to sort the cohorts by how much change in VTS they likely exhibited. The cohort with the largest increase (i.e. 0.16; February 2009) in the ratio should have experienced the greatest decrease in the number of survivors of VTS among male singletons in gestation. The cohort with the largest decrease in the ratio (i.e. –0.17; December 1997) should have experienced the greatest increase in the number of survivors of VTS among male singletons in gestation. Second, we multiplied the largest increase (i.e. 0.16) and decrease (i.e. –0.17) by the estimated coefficient (i.e. –27.3) for the ratio of male to female twin births at 37 weeks gestation. The products implied that change in male survivors of VTS could have accounted for a maximum increase or decrease of about 4.5 male infant deaths. So, the 63 infant deaths among male singletons, born at 40 weeks gestation, to the cohort conceived in February 2009 were 4.5, or about 7.3%, *fewer* than would have occurred had VTS survivors in the cohort been the same as in the cohort conceived in January. Obversely, the 97 infant deaths among male singletons born at 40 weeks gestation from the cohort conceived in December 1997 were 4.5, or about 4.7%, *more* than would have occurred had VTS survivors in the cohort been the same as in the cohort conceived in November 1998. Based on the above calculations, we infer that the contribution of survivors of VTS to temporal variation in infant mortality among male singletons born at 40 weeks gestation, the hardiest of male infants, ranged from a decrease of about 7% to an increase of about 5% over our 276 conception cohorts.

As with all observational tests of association, an unspecified “third variable” that decreased the sex ratio of twins born at 37 weeks gestation while increasing infant mortality among male singletons born at 40 weeks gestation may have induced our results. We note, however, that our design controls any unspecified confounder that correlates with the size of the population at risk, affects mortality in female singletons born in the 40th week of gestation, or exhibits autocorrelation. We further note that we used “E-value” testing [44] to assess the likelihood that an unspecified confounder could empirically “explain away” our finding. We estimated an E-value of 9.34 (CI = 3.33). This value implies that the sex ratio of twins born at 37 weeks gestation from conception cohorts exposed to the unspecified confounder would be 1/9 that among twins born at 37 weeks from unexposed cohorts. It further implies an average of 9 more infant deaths among males born at 40 weeks gestation from hypothetically exposed cohorts than among those born at 40 weeks from unexposed cohorts. Given an average of 69 male deaths for all cohorts, this represents a greater than 13% increase. We know of no phenomenon that could have such extreme and opposing effects on the sex ratio of twins and infant mortality among singletons.

## DISCUSSION

We set out to test the hypothesis, implied by an evolutionary perspective on heretofore separate literatures describing VTS and infant frailty, that the male-to-female ratio of twins born from conception cohorts at the 37th week of gestation will correlate inversely with infant mortality among male singletons born in the 40th week of gestation from the same cohorts. We found that association.

Further research should test the proposition that previously reported associations between population stressors and infant mortality might arise from VTS. The epidemiologic literature includes reports, for example, that contracting economies increase infant mortality [45–47]. Adding the twin sex ratio to the predictors in such tests and comparing its contribution to explained variance to contributions made by other predictors could shed light on the role, if detectable, VTS plays in the association between prevailing economies and infant mortality.

Further research should also test the possibility that the experiences of subgroups defined by race/ethnicity, geography, or available health care modify the results we report. Data from the US has, for example, supported the hypothesized contribution of VTS to differences in birthweight between infants born to Non-Hispanic Black and white women [48].

We believe our findings make several contributions to basic and applied scholarship concerned with infant mortality in the US. First, we offer a novel indicator (i.e. the ratio of male to female twins born at 37 complete weeks of gestation) of the incidence of VTS in monthly conception cohorts. For reasons described above, researchers cannot directly observe the incidence of VTS and must, therefore, infer its variation over cohorts from its observable sequelae. Our indicator assumes VTS is one of several mechanisms thought to spontaneously abort fetuses that, if born, would unlikely survive in the prevailing environment. The literature describing the causes and effects of selection *in utero*, therefore, supports our indicator [25].

Second, we believe our argument and findings suggest a mechanism that likely contributes to the high rates of infant mortality among groups in the US subjected to the adversity of relative poverty and or racism [49]. Assuming that pregnant persons in these groups suffer relatively high rates of VTS, singletons born to them will include comparatively many VTS survivors who, in turn, contribute to relatively high rates of infant mortality.

Third, our arguments and findings suggest that earlier detection and better tracking of twin gestations would allow clearer accounting of infant mortality by separating survivors of VTS, a population at relatively high risk of infant mortality, from true singletons. Our findings also suggest, however, that reducing fetal loss among twins would have complex effects on infant mortality. Twin fetuses saved from spontaneous abortion or still birth will likely have a comparatively high rate of infant mortality compared to other twins thereby increasing the rate among twins in their conception cohorts. VTS, however, will likely be less common implying fewer male singleton survivors of the Syndrome. This, in turn, implies lower infant mortality among male singletons. The net effect on infant mortality in the entire cohort may, therefore, be small despite inevitable reports of increases among twins and decreases among singletons.

Fourth, epidemiologists [12] have used data describing the characteristics of twins to estimate the relative contributions of fetal defects and anomalous development to infant mortality in singletons. These estimations assume that twins and singletons gestate as separate populations. We believe our findings call this assumption, and the calculations based on them, into question.

Fifth, our arguments and findings also call into question the characterization of developmental anomalies as “confounders” difficult to measure in fetal cohorts because the defects appear idiopathic or without signal [10, 12]. We show that VTS, a well-characterized mechanism known to cause anomalous development among its singleton survivors, can not only be measured, albeit indirectly, in conception cohorts but also increases infant mortality in those cohorts. We also suggest that researchers can understand VTS as a form of selection *in utero*. As such, its causes and effects are explicable from the logic of natural selection—perhaps the most widely understood heuristic in the biological sciences.

## Data Availability

We will make all de-identified data publicly available on our GitHub repository.
